# Computation and analysis of temporal betweenness in a knowledge mobilization network

**DOI:** 10.1186/s40649-017-0041-7

**Published:** 2017-07-10

**Authors:** Amir Afrasiabi Rad, Paola Flocchini, Joanne Gaudet

**Affiliations:** 1 School of Electrical Engineering and Computer Science, University of Ottawa, Ottawa, Ontario Canada; 2 Alpen Path Solutions Inc., Ottawa, Ontario Canada

**Keywords:** Time-varying graphs, Temporal betweenness, Dynamic networks, Temporal analysis, Social networks

## Abstract

**Background:**

Highly dynamic social networks, where connectivity continuously changes in time, are becoming more and more pervasive. Knowledge mobilization, which refers to the use of knowledge toward the achievement of goals, is one of the many examples of dynamic social networks. Despite the wide use and extensive study of dynamic networks, their temporal component is often neglected in social network analysis, and statistical measures are usually performed on static network representations. As a result, measures of importance (like betweenness centrality) typically do not reveal the temporal role of the entities involved. Our goal is to contribute to fill this limitation by proposing a form of temporal betweenness measure (foremost betweenness).

**Methods:**

Our method is analytical as well as experimental: we design an algorithm to compute foremost betweenness, and we apply it to a case study to analyze a knowledge mobilization network.

**Results:**

We propose a form of temporal betweenness measure (foremost betweenness) to analyze a knowledge mobilization network and we introduce, for the first time, an algorithm to compute exact foremost betweenness. We then show that this measure, which explicitly takes time into account, allows us to detect centrality roles that were completely hidden in the classical statistical analysis. In particular, we uncover nodes whose static centrality was negligible, but whose temporal role might instead be important to accelerate mobilization flow in the network. We also observe the reverse behavior by detecting nodes with high static centrality, whose role as temporal bridges is instead very low.

**Conclusion:**

In this paper, we focus on a form of temporal betweenness designed to detect accelerators in dynamic networks. By revealing potentially important temporal roles, this study is a first step toward a better understanding of the impact of time in social networks and opens the road to further investigation.

## Background

Highly dynamic networks are networks where connectivity changes in time and connection patterns display possibly complex dynamics. Such networks are more and more pervasive in everyday life and the study of their properties is the object of extensive investigation in a wide range of very different contexts. Some of these contexts are typically studied in computer science, such as wireless, ad hoc networks, transportation, vehicular networks, satellites, military, and robotic networks (e.g., see [1–6]), while others belong to totally different disciplines. This is the case, for example, of the nervous system, livestock trade, epidemiological networks, and multiple forms of social networks (e.g., see [7–12]). Clearly, while being different in many ways, these domains display common features; a *time-varying graph* (TVG) is a model that formalizes highly dynamic networks encompassing the above contexts into a unique framework and emphasizes their temporal nature [13].

*Knowledge mobilization* (KM) refers to the use of knowledge toward the achievement of goals [14]. Scientists, for example, use published papers to produce new knowledge in further publications to reach professional goals. In contrast, patient groups can use scientific knowledge to help foster change in patient practices, and corporations can use scientific knowledge to reach financial goals. Recently, researchers have started to analyze knowledge mobilization networks (KMN) using a social network analysis (SNA) approach (e.g., see [15–20]). In particular, [19] proposed a novel approach where a heterogeneous network composed of a main class of actors subdivided into three subtypes (individual human and non-human actors, organizational actors, and non-human mobilization actors) associated according to one relation, knowledge mobilization (a mobilization-network approach). Data covered a 7-year period with static networks for each year. The mobilization network was analyzed using classical SNA measures (e.g., node centrality measures, path length, density) to produce understanding for KM using insights from network structure and actor roles [19].

The KM SNA studies mentioned above, however, lack a fundamental component: in fact, their analysis is based on a static representation of KM networks, incapable of sufficiently accounting for the time of appearance and disappearance of relations between actors beyond static longitudinal analysis. Indeed, incorporating the temporal component into analysis is a challenging task, but it is undoubtedly a critical one, because time is an essential feature of these networks. Temporal analysis of dynamic graphs is in fact an important and extensively studied area of research (e.g., see [21–27]), but there is still much to be discovered. In particular, most temporal studies simply consider network dynamics in successive static snapshots, thus capturing only a partial temporal component by observing how static parameters evolve in time while the network changes. Moreover, very little work has been dedicated to empirically evaluating the usefulness of metrics in time (e.g., see [28, 29]).

In this paper, we represent KMN by TVGs and we propose to analyze them in a truly temporal setting. We design a deterministic algorithm to compute a form of temporal betweenness in time-varying graphs (*foremost betweenness*) that measures centrality of nodes in terms of how often they lie within temporal paths with the earliest arrival. We then provide, for the first time on a real data set, an empirical indication for the effectiveness of foremost betweenness. In particular, we focus on data extracted from [19], here referred to as *Knowledge-Net*. We first consider static snapshots of Knowledge-Net corresponding to the 7 years of its existence, and by studying the classical centrality measures in those time intervals, we provide rudimentary indications of the networks’ temporal behavior. To gain a finer temporal understanding, we then concentrate on *temporal betweenness* following a totally different approach. Instead of simply observing the static network over consecutive time intervals, we focus on the TVG that represent Knowledge-Net and we compute foremost betweenness, explicitly and globally taking time into account. We compare the temporal results that we obtain with classical static betweenness measures to gain insights into the impact that time has on the network structure and actor roles. We notice that, while many actors maintain the same role in static and dynamic analysis, some display striking differences. In particular, we observe the emergence of important actors that remained invisible in static analysis, and we advance explanations for these. Results show that the form of temporal betweenness we apply is effective at highlighting the role of nodes whose importance has a temporal nature (e.g., nodes that contribute to mobilization acceleration).

A limitation of our algorithm is its applicability to small networks. In fact, any deterministic solution to the computation of foremost betweenness is inevitably very costly and, when faced with large networks, it is feasible to apply it only on small components. This research opens the road to the design of approximate variations of the algorithm so to make it applicable to larger scenarios, as well as to the study of other temporal measures designed for TVGs.

## Time-varying graphs

### Definition

Time-varying graphs are graphs whose structure varies over time. Following [13], a time-varying graph (TVG) is defined as a quintuple $\mathcal{G} = {(V,E,\mathcal{T},\rho ,\zeta )}$, where *V* is a finite set of nodes and $E \subseteq V \times V$ is a finite set edges. The graph is considered within a finite time span $\mathcal {T}\subseteq \mathbb {T}$, called lifetime of the system. $\rho {:}\, E \times \mathcal{T} \rightarrow \{0,1\}$ is the edge presence function, which indicates whether a given edge is available at a given time; $\zeta {:} \,E \times \mathcal{T} \rightarrow {\mathbb T}$ is the latency function, which indicates the time it takes to cross a given edge if starting at a given date. The model may, of course, be extended by defining the vertex presence function $(\psi {:}\,V\times \mathcal {T}\rightarrow \{0,1\})$, and vertex latency function $(\phi {:}\,V\times \mathcal {T}\rightarrow \{0,1\})$. The footprint of $\mathcal{G}$ is a static graph composed by the union of all nodes and edges ever appearing during the lifetime $\mathbb {T}$.

### Journeys

A journey route *R* in a TVG $\mathcal {G}$ is a walk in *G* defined as a sequence of edges $\{e_1,e_2,\ldots ,e_k\}$. A journey $\mathcal {J}$, then, is a temporal walk in $\mathcal {G}$ comprising the sequence of ordered pairs $\{(e_1,t_1),(e_2,t_2),\ldots,$ $(e_k,t_k)\}$ if and only if $\rho (e_i,t_i)=1$ and $t_{i+1}\ge t_i+\zeta (e_i,t_i)$ for all $i<k$. Every journey has a departure $(\mathcal {J})$ and an arrival $(\mathcal {J})$ that refer to journey’s starting time $t_1$ and its finish time $t_k+\zeta (e_k,t_k)$, respectively. Journeys are divided into three classes based on their variations based on the temporal and topological distance [30]. Journeys that have the earliest arrival times are called *foremost* journeys, journeys with the smallest topological distance are referred to as the *shortest* journeys, while the journey that takes the smallest amount of time is called the *fastest*. Moreover, we call *foremost increasing journeys* the ones whose route $\{e_1,e_2, \ldots , e_k\}$ is such that *birth-date* $(e_i) \le$ *birth-date* $(e_{i+1})$.

### Temporal betweenness

Betweenness is a classic measure of centrality extensively investigated in the context of social network analysis. The betweenness of a node $v\in V$ in a static graph $G=(V,E)$ is defined as follows:

1 $$\begin{aligned} B(v) =\sum _{u\ne w\ne v\in V}\frac{|P(u,w,v)|}{|P (u,w)|}, \end{aligned}$$

where |*P*(*u*, *w*)| is the number of shortest paths from *u* to *w* in *G*, and |*P*(*u*, *w*, *v*)| is the number of those passing through *v*. Even if static betweenness is “atemporal,” we denote here by $B(v)^ \mathcal {T}$ the static betweenness of a node *v* in a system whose lifetime is $\mathcal {T}$. Typically, vertices with high betweenness centrality direct a greater flow and, thus, have a high load placed on them, which is considered as an indicator for their importance as potential gatekeepers in the network.

While betweenness in static graphs is based on the notion of the shortest path, its temporal version can be extended into three different measures to consider the shortest, foremost, and fastest journeys for a given lifetime $\mathcal {T}$ [25].

In this paper, we consider foremost betweenness. Nodes with a high foremost betweenness values do not simply act as gatekeepers of flow, like their static counterparts. In fact, they direct the flow that conveys a message in an *earliest* transmission fashion. In other words, if the message transmission takes the path from foremost between nodes, such nodes provide a means to transmit the message in a more timely manner to all other nodes in the graph compared to the nodes that have lower foremost centrality. Thus, intuitively, they provide some form of “acceleration” in the flow of information.

Foremost betweenness $TB^ \mathcal {T}_\mathcal{F} (v)$ for node *v* with lifetime $\mathcal {T}$ is here defined as follows:

2 $$\begin{aligned} TB^ \mathcal {T}_\mathcal{F} (v) = {n(v) \over n} \sum _{u\ne w\ne v\in V}\frac{|\mathcal {F}^\mathcal {T} (u,w,v)|}{|\mathcal {F}^ \mathcal {T}(u,w)|}, \end{aligned}$$

where $|\mathcal {F}^ \mathcal {T}(u,w)|$ is the number of foremost *journey routes* between *u* and *w* during time frame $\mathcal {T}$ and $|\mathcal {F}^ \mathcal {T}(u,w,v)|$ is the number of the ones passing through *v* in the same time frame; *n* is the total number of nodes, and *n*(*v*) is the number of nodes in the connected component to which *v* belongs. The factor $n(v) \over n$ is an adjustment coefficient to take into account possible network disconnections. In fact, it makes the betweenness of a node depend on the actual size of the connected component to which the node belongs, thus avoiding anomalous situations where a node in a very small component could be otherwise perceived as globally central. This would be the case, for example, of the center *v* of a small component in the shape of a star, where *v* would have maximum global betweenness while its central role is applied only to a very small portion of the overall network.

## Computing foremost betweenness 

The computation of betweenness centrality in static graphs can be done quite efficiently. Several approaches exist in the literature (e.g., see [31–35]) proposing either polynomial deterministic solutions or approximate ones for a variety of different graphs. Computing shortest-path betweenness in TVG can also be done in polynomial time, for example by adapting the algorithms described in [26, 30]. The situation is rather different in the case of foremost betweenness, for which no algorithm has been proposed so far. In fact, it is easy to see that there exist TVGs where counting all foremost journeys or journey routes between two vertices is #P-complete, which means that no polynomial-time algorithm is known.

Consider, for example, TVGs where edges always exist (note that a static graph is a particular TVG) and latency is zero. In such a case, any journey between any pair of nodes is a foremost journey. Counting all of them is then equivalent to counting all paths between them, which is a #P-complete problem (see [36]). In general, it is then unavoidable to have worst-case exponential algorithms to compute foremost betweenness in an arbitrary TVG.

In this section, we first focus on foremost betweenness based on journey routes in the general setting (Algorithm 1). We then focus on foremost betweenness for special TVGs with zero latency and instant edges (Algorithm 2), which correspond to the characteristics of the knowledge mobilization network that we analyze in "Knowledge-Net". Note that each solution has the same worst-case time complexity, linear in the number of nodes in all the journey routes in the TVG, which can clearly be exponential. The advantages of the algorithm designed for the special temporal condition of instant edges and zero latency are mainly practical. In fact, the worst-case complexities are the same, but the execution time is better for our particular dataset.

### A general algorithm

In this section, we describe an algorithm for counting all journey routes from a given node to all the other nodes in the TVG, passing through any possible intermediate node. This module is at the basis of the computation of foremost betweenness.

We start by introducing some notations and functions used in the algorithm. Given an edge (*x*, *y*), let function arriv(*x*, *y*, *t*) return the arrival time to *y*, leaving *x* at time *t*. Given a time-stamped journey $\pi$, with an abuse of notation, let us indicate by arriv$(\pi )$ the arrival time at the last node of $\pi$. The foremost arrival time in *G* to any node *v* from a given source *s* can be computed using the Algorithm from [30]. Let foremost(*s*, *v*) denote such a time.

We are now ready to describe the algorithm. The input of Algorithm CountFormemostJRoutes is a pair (*G*, *s*), where $$G=(V,E) $$ is a TVG and *s* is a starting node. The algorithm returns a matrix Count$$_s[x,y]$$, for all $$x,y \in V$$ containing the number of foremost journeys from *s* to *y* passing through *x* (note that Count$$_s[x,x]$$ denotes the number of foremost journeys from *s* to *x*).

The counting algorithm is simple and it is based on multiple Depth-First Search (DFS) traversals. It consists of visiting every journey route of *G* starting from *s*, incrementing the appropriate counters every time a newly encountered journey is foremost. We remind that a node can reappear more than once in a journey route, with various occurrences corresponding to different times. This means that we need to store the time when a node is visited in the journey route so that, if it is visited again, we can determine whether the subsequent visit corresponds to a later time and thus the node has to be considered again. Note that this is the main difference with respect to a DFS in a static graph, where instead every node is visited exactly once.

To perform the traversal managing multiple visits (corresponding to different traversal times), we use two stacks: *Path* and *S*, where *Path* contains the nodes corresponding to the journey currently under visit and *S* contains the edges to be visited. In both *Path* and *S*, we store also time-stamps, to register the time of the first visit of nodes in *Path* and the time for the future visits of edges in *S*. If a node happens to be revisited at a later time, in fact, it is treated as a new node.

The traversal starts as a typical DFS, pushing the incident edges of the source *s* onto stack *S* with their arrival times in these journeys (lines 4–6). The nodes corresponding to the current journey under visit are kept in the second stack *Path* (these nodes are implicitly marked *visited*), initially containing only the source. When considering the next candidate edge (*x*, *y*) to visit (line 8), we may be continuing the current journey (if the top of stack *Path* contains *x*) or we may have backtracked to some previous nodes (if the top is different from *x*). In this last case, the content of *Path* is updated to reflect the backtracking (lines 9–11). After visiting a node *y* (line 16), the DFS continues pushing on *S* the edges incident to *y* that are feasible with the current journey under visit (i.e., the edges whose target is not already in *Path*, and whose latest traversal time is greater than or equal to the earliest arrival time at *y*) (lines 17–19). The *if* clause at line 20 checks whether the discovered journey is foremost and updates the corresponding counters.

In other words, as soon as a journey $\pi = [(x_0,x_1),(x_1,x_2), \ldots ,(x_{k-1},x_k)]$ is encountered in the traversal, Count$[x_i,x_k]$, $i\le k$ is updated only if $\pi$ is a foremost journey, and, regardless of it being foremost, the traversal continues pushing on the stack the edges incident to $x_k$ that are temporally feasible with $\pi$. Whenever backtracking is performed, however, the already visited nodes on the backtracking path are popped from *Path* (thus implicitly remarked *unvisited*) in such a way that they can be revisited as part of different journey routes, not explored yet.

#### Observations on complexity

The running time of Algorithm CountFormemostJRoutes is linear in the number of nodes belonging to different foremost journeys, because it traverses each one of them. However, depending on the structure of the TVG, such a number could be exponential, thus an overall exponential worst-case complexity.

More precisely, let $\mu _s$ be the number of foremost journeys from a source node *s* to all the other nodes in $\mathcal{G}$, $n(\mu _s)$ be the number of nodes belonging to those journeys, and *n* the number of nodes of $\mathcal{G}$. Moreover, let $\mu$ and $n(\mu )$ be, respectively, the overall number of foremost journeys in $\mathcal{G}$ and the overall number of nodes in those journeys. The algorithm to count all foremost journeys from *s* to all the other nodes traverses every foremost journey from the source to any other node, and it performs an update for every visited node in each foremost journey that it encounters. Thus, its time complexity is $O(n(\mu _s))$. To compute foremost betweenness, the algorithm has to be repeated for every possible source, thus traversing every possible foremost journey in $\mathcal{G}$ for a total time complexity of $O(n(\mu ))$. Since $n(\mu )$ could be exponential in *n*, we have a worst-case exponential complexity in the size of the network. Note that the high cost is inevitable for any deterministic algorithm to compute foremost betweenness.

### Algorithm for KnowledgeNet

Algorithm 1 is applicable to a general TVG. We now consider a very special type of TVG with specific temporal restrictions that correspond to the type of network that we analyze in this paper. One such peculiarity is given by *instant edges* (edges that appear only during a unique time interval). Another characteristic is *zero latency* (i.e., edges that can be traversed instantaneously). Finally, in this setting, we base betweenness computation on increasing journey routes.

We then describe a variation of the general algorithm specifically designed for those conditions (instant edges with zero latency), and we compute foremost betweenness applying the foremost betweenness formula restricted to foremost increasing journeys.

Given a TVG $G=(V,E)$, since we assume the presence of instant edges, we can divide time in consecutive intervals $I_1, I_2, \ldots , I_k$ corresponding to *k* snapshots $G_1, G_2, \ldots G_k$ ($G_i=(V_i,E_i)$), in such a way that $(x,y)\in E_i$ implies that $(x,y)\not \in E_j$ for $j\ne i$. Furthermore, we know by $\zeta =0$ that an edge can be traversed in zero time.

The key idea that can be applied to this very special structure is based on the observation that, given a foremost route $\pi _{x,y}$ from *x* to *y* with edges in time intervals $I_j$, provided that $j>i$ and *j* appears immediately after *i*, and given any journey route $\pi '_{s,x}$ from *s* to *x* with edges only in $I_i$, the concatenation of $\pi '_{s,x}$ and $\pi _{x,y}$ is a foremost route from *s* to *y*, passing through *x*.

This observation leads to the design of an algorithm that starts by counting the foremost routes belonging to the last snapshot $G_k$ only, and proceeds backwards using the information already computed. More precisely, when considering snapshot $G_i$ from a source *s*, the goal is to count all foremost routes involving only edges in $\cup _ {j\ge i} E_j$ (i.e., with time intervals in $\cup _ {j\ge i} I_j$), and when doing so, all the foremost routes involving only edges strictly in the “future” (i.e., time intervals $\cup _ {j>i} I_j$) have been already calculated for any pair of nodes. The already computed information is used when processing snapshot $G_i$ in a dynamic programming fashion.

As for Algorithm 1, the input of Algorithm 2 is a pair: a snapshot $G_i$ and a starting node *s*. The algorithm returns an array, Count$_{s}[u,v]$, where Count$_{s}[u,v]$ for all $u,v \in V$ contains the number of foremost journeys from *s* to *u* passing through *v* counted so far (i.e., considering only edges in $\cup _{j\ge i} E_j$).

The actual counting algorithm on snapshot $G_i$ is a modified version of Algorithm 1, still based on Depth-First Search (DFS) traversal. Lines 2–11 are exactly the same as in Algorithm 1, except that here we do not need to keep track of the arrival time for each edge, as we run Algorithm 2 in a single snapshot and the latency for edges is zero.

In line 13, we examine whether the target of the current edge *y* has already been visited or not. If it has not been visited already, it either falls in the current snapshot, or it flows into the next snapshot.

In the case where *y* stays in the current snapshot (lines 15–22), we push its adjacent nodes into the stack *S* and determine whether the route ending at *y* is foremost. If a foremost route is discovered at *y*, we update Count$_{s}[z,v]$ by incrementing its value for all $z \in Path$ (*z* being the node that falls on the journey route from *s* to *y*).

If instead it is not a foremost route in the current interval (lines 23–25), meaning that *y* is a node that existed in the “future,” a special update is performed using the data already calculated for the “future snapshots.”

More precisely, when a journey route (in this case a foremost journey route) from *s* to *x* ($s\leadsto x$) is a prefix of a journey route $x\leadsto y$ at a later time snapshot, we perform a procedure called SpecialCount (Algorithm 3). The special count procedure involves aggregating the values of Count$_s[v,x]$ with Count$_x[v',y]$, for all nodes (resp. *v*, $v'$) occurring in the journey routes between *s* and *x* and between *x* and *y* (see Algorithm 3). Algorithm 3 simply calculates the product of the number of foremost journeys between two routes $s\leadsto x$, and $x\leadsto y$, if they do not share any vertex (lines 4–9). If instead they share some vertex *v*, the calculation is slightly more complicated: let *a* be the number of foremost journeys from *s* to *y* where *v* is visited at least once on the route between *x* and *y*; let *b* be the number of foremost journeys from *s* to *y* where *v* is visited at least once on the route between *s* and *x*; and let *c* be the sum of *a* and *b*. *c* represents the number of all foremost journeys from *s* to *y* that pass through *v*. However, *c* counts the journey route passing through *v* multiple times if *v* happened to exist in both Count$_s[v,x]$ and Count$_x[v,y]$, and we need to remove such multiple counting of journeys, which is done along with the update to Count$_s[v,y]$ in line 13.

#### Observations on Complexity

The worst-case time complexity of Algorithm 2, CountAllZeroLatency, is the same as the one of the general algorithm, CountFormemostJRoutes. In our network, however, it performed better than Algorithm 1. We try to explain below the reasons for this.

Algorithm 2 has to be executed in anti-chronological order of the different snapshots, starting from the last one, since it uses the previously calculated results in the computation of the new results. This approach is amenable to concurrent computations. In fact, since the graph is divided into independent snapshots, the number of all journeys can be computed separately for each snapshot, and the result of the calculation can be aggregated at the end. This has the advantage of eliminating all the special updates from the first part of the algorithm (while detecting all the journey routes), and deferring them to the second part (when aggregating all the information for the final update). Thus, instead of performing the special count at each level, we can postpone it to the last step of the algorithm, and loop once through all the collected counts with hard-coded intervals in the loop.

While not being advantageous in worst-case scenarios, this strategy results in a more efficient solution from a practical point of view. Still, the algorithm is very costly, even in such a small network (KnowledgeNet has 366 vertices and 750 edges) and it did run in almost a month when implemented in C++ with a machine with 40 cores and 1TB RAM.

## Knowledge-Net

*Knowledge-Net* is a heterogeneous network where nodes represent human and non-human actors (researchers, projects, conference venues, papers, presentations, laboratories) and edges represent knowledge mobilization between two actors. The network was collected for a period of 7 years [19]. Once an entity or a connection is created, it remains in the system for the entire period of the analysis.

Table 1 provides a description of the *Knowledge-Net* dataset. The dataset consists of 366 vertices and 750 edges in 2011. The numbers of entities and connections vary over time starting from only 10 vertices and 14 edges in 2005 and accumulating to the final network year in 2011. *Knowledge-Net* is mainly composed of non-human actors, 272 in total (non-human mobilization actors, NHMA, non-human individual actors, NHIA, and organizational actors, OA), in relation with 94 human actors (HA). Human actors include principle investigators (PI), highly qualified personnel (HQP), and collaborators (CO). It is through mobilization actors (NHMA) that individual, organizational actors and mobilization actors associate and mobilize knowledge to reach goals. For example, scientists mobilize knowledge through articles where not all contributing authors might be in relation with all other authors, yet all relate with the publication [19]. These non-human mobilization actors make up the bulk of the network including conference venues, presentations (invited oral, non-invited oral, and poster), articles, journals, laboratories, research projects, websites, and theses.

According to an interpretation of the *the Actor-Network Theory* [37], the nature/ type/characteristics of the mobilizer nodes have no interference with their role as a mobilizer. Following this interpretation, we consider that knowledge mobilization is beyond the role and nature of the nodes and we treat KnowledgeNet as a homogeneous network of *knowledge mobilizers*. All nodes of this network have the same function as *knowledge mobilizer* despite the fact that they might be quite different from each other from the view point of nature, type, and/or characteristics.

**Table 1:** *Knowledge-Net* data set with characteristics of actors and their roles at different times

Start	Duration	#Nodes	#Edges	Granularity
2005	7 Years	366	750	1 Year

Actor type	2005	2006	2007	2008	2009	2010	2011
HIA	3	22	27	46	51	76	94
NHIA	0	3	6	9	9	9	15
NHMA	7	25	43	87	132	194	248
OA	0	5	5	9	9	9	2
Total	10	55	81	151	201	288	366

Classical statistical parameters have been calculated for Knowledge-Net, representing it as a static graph where the time of appearance of nodes and edges did not hold any particular meaning. In doing so, several interesting observations were made regarding the centrality of certain nodes as knowledge mobilizers and the presence of communities [19]. In particular, all actor types increased in number over the 7 years indicating a rise in new mobilization relations over time. Although non-human individual actor absolute numbers remained small (ranging from 3 in 2006 to 15 in 2011), these actors were critical to making visible tacit (non-codified) knowledge mobilization from around the world (mostly laboratory material sharing, including from organizations and universities in the USA, from Norway, and from Canadian universities). Finally, embedded in human individual actor counts were individuals that the laboratory acknowledged in peer-reviewed papers, thus making further tacit and explicit knowledge mobilization visible.

**Fig. 1:**
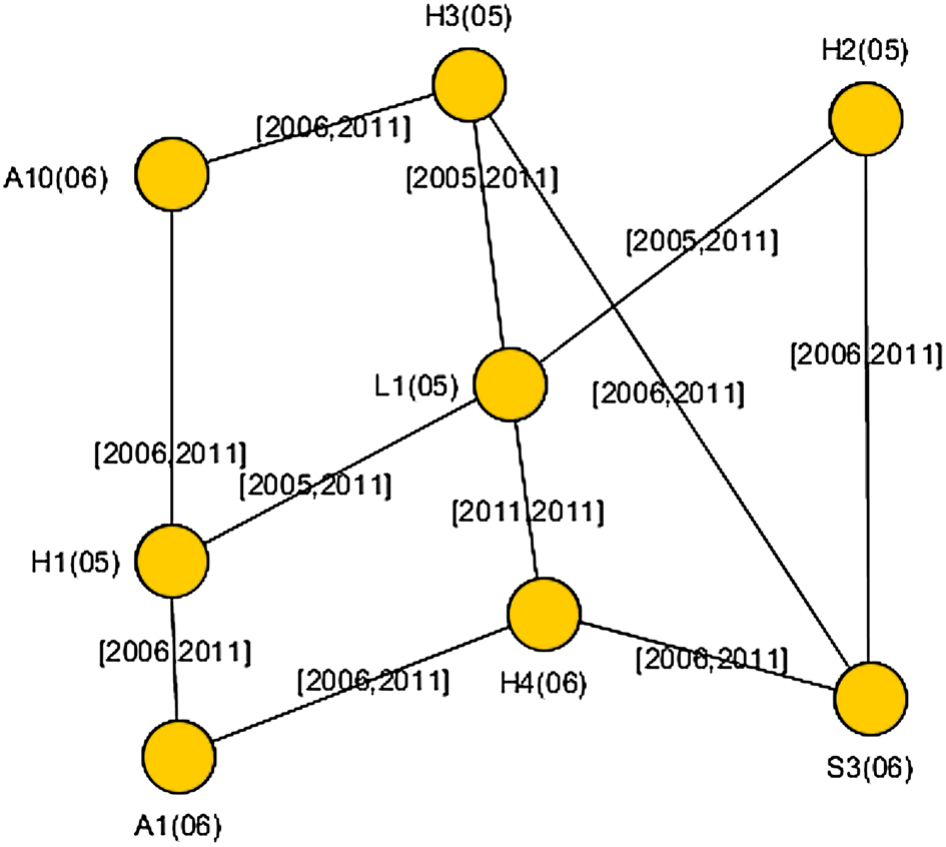
A small portion of Knowledge-Net represented as a TVG

When representing *Knowledge-Net* as a TVG, we notice that the latency $\zeta$ is always zero, as an edge represents a relationship and its creation does not involve any delay; moreover, edges and nodes exist from their creation (their birth-date) to the end of the system lifetime. Let *birth-date*(*e*) denote the year when edge *e* is created. An example of a small portion of *Knowledge-Net* represented as a TVG is shown in Fig. 1.

We also notice that, due to zero latency, edges spanning only one interval, and to the fact that edges never disappear once created, any shortest journey route in $\mathcal{G}$ is equivalent to a shortest path on the static graph corresponding to its footprint; moreover, the notion of fastest journey does not have much meaning in this context, because on any route corresponding to a journey, there would be a fastest one. On the other hand, the notion of foremost journey, and in particular of foremost increasing journey, is extremely relevant as it describes timely mobilization flow, i.e., flow that arrives at a node as early as possible.

Note that in this setting the computation of foremost betweenness can be performed using Algorithm 2 introduced in the previous section.

## Study of KnowledgeNet

### Analysis on consecutive snapshots

To provide more clear statistics on the Knowledge-Net dataset and a ground for better understanding of temporal metrics, we first calculated classical statistical measures (e.g., node centrality measures, path length, density) on the seven static graphs, corresponding to the 7 years of study. The average for each value for the graphs is calculated to represent a benchmark on how the rank for each node is compared to others.

**Table 2:** Some static statistical parameters calculated for successive snapshots

	2005	2006	2007	2008	2009	2010	2011
Ave. degree	1.40	1.32	1.63	1.84	1.98	2.02	2.04
Diameter	4	5	5	6	6	6	6
Density	0.31	0.04	0.04	0.02	0.02	0.01	0.01
#Communities	4	3	6	8	8	15	12
Modularity	0.17	0.52	0.46	0.47	0.46	0.54	0.54
Ave. clustering coefficient	0.41	0.06	0.21	0.22	0.20	0.24	0.23
Ave. path length	2.04	3.04	3.06	3.26	3.34	3.46	3.50
Ave. normalized closeness	0.51	0.33	0.33	0.31	0.30	0.29	0.29
Ave. eccentricity	3.10	4.41	4.40	4.70	4.80	4.83	4.83
Ave. betweenness	4.70	58.36	83.53	169.70	234.89	354.23	456.18
Ave. normalized betweenness	0.13	0.03	0.02	0.01	0.01	$\approx$ 0	$\approx$ 0
Ave. page rank	0.10	0.01	0.01	$\approx$ 0	$\approx$ 0	$\approx$ 0	$\approx$ 0
Ave. eigenvector	0.52	0.19	0.15	0.10	0.09	0.07	0.05

The statistical data presented in Table 2 provide valuable information about the graph. The steady decrease in the centrality values (normalized in the [0,1] range) confirms that the network growth is not symmetric, so the centrality values have long tails. According to Hanneman and Riddle [38], we should expect a high value of betweenness in dense graphs due to the fact that it is highly possible that a path crosses every node. Meanwhile, when the betweenness values are normalized, they become low if all of the betweenness values are close to each other. Thus, the high value of betweenness (in the range of hundreds), and the low value of its normalized counterpart (close to zero) in Knowledge-Net, indicates that the graph is either dense or is coupled in a way that there is a large number of shortest paths between any two arbitrary vertices. The graph is not dense as it is confirmed by the highest density metric of six. Therefore, the high number of shortest paths in the graphs caused the betweenness for most vertices to be similar and quite low when compared to the ones of nodes with the highest betweenness. Low average path length (highest being 3.50) is a sign that the network presents small-world characteristics and the knowledge mobilization to the whole network is expected to be conducted only in a few hops. Meanwhile, the decreasing graph density (from 0.3 to 0.1) along with the increasing average degree (from 1.4 to 2.04) represents the slow growth in the number of edges compared to the number of nodes. Escalation in the number of communities (by 8 communities) with an increase in graph modularity metrics (from 0.17 to 0.54) shows that the knowledge mobilization actors tend to form communities as time progresses. As the normalized average betweenness decreases steadily, it might be concluded that a few vertices in each community play the role of mediators and create the link between communities.

Apart from these general observations, a static analysis of consecutive snapshots does not provide temporal understanding. For example, it does not reflect which entities engage in knowledge mobilization in a timely fashion, e.g., by facilitating fast mobilization, or slowing mobilization flow.

To tackle some of these questions, we represent *Knowledge-Net* as a TVG and we propose to study it by employing a form of temporal betweenness that makes use of time in an explicit manner.

### Foremost betweenness of Knowledge-Net

In this section, we focus on *Knowledge-Net*, and we study $TB^ \mathcal {T}_\mathcal{F} (v)$ for all *v*. Nodes are ranked according to their betweenness values and their ranks are compared with the ones obtained calculating their static betweenness $B^ \mathcal {T}(v)$ in the same time frame. Given the different meaning of those two measures, we expect to see the emergence of different behaviors, and, in particular, we hope to be able to detect nodes with important temporal roles that were left undetected in the static analysis.

#### Foremost Betweenness during the lifetime of the system

Table 3 shows the temporally ranked actors accompanied by their static ranks, and the high-ranked static actors with their temporal ranks, both with lifetime $\mathcal {T}=$ [2005–2011]. In our naming convention, an actor named *Xi*(*yy*) is of type *X*, birth-date *yy*, and it is indexed by *i*; types are abbreviated as follows: *H* (human), *L* (Lab), *A* (article), *C* (conference), *J* (journal), *P* (project), *C* (paper citing a publication), *I* (invited oral presentation), and *O* (oral presentation). Note that only the nodes whose betweenness has a significant value are considered, in fact betweenness values tend to lose their importance, especially when the differences in the values of two consecutive ranks are very small [34].

**Table 3:** List of the highest ranked actors according to temporal (resp. static) betweenness, accompanied by the corresponding static (resp. temporal) rank in lifetime [2005–2011]

Temporal to static			Static to temporal		
Actor	Temporal rank	Static rank	Actor	Static rank	Temporal rank
L1(05)	1	1	L1(05)	1	1
H1(05)	2	2	H1(05)	2	2
A1(06)	3	3	A1(06)	3	3
A2(08)	4	4	A2(08)	4	4
P1(06)	5	8	A5(08)	5	12
A3(07)	6	9	A4(09)	6	7
A4(09)	7	6	P2(08)	7	9
S1(10)	8	115	P1(06)	8	5
P2(08)	9	7	A3(07)	9	6
J1(06)	10	160	P3(10)	10	17
C1(07)	11	223	A6(11)	11	18
A5(08)	12	5	A8(09)	12	36
I1(09)	13	28	P4(10)	13	22
O1(05)	14	45	P5(11)	14	27
S2(05)	15	46	H2(05)	15	44
I2(05)	16	47	A7(09)	16	21
P3(10)	17	10	A9(10)	17	31
A6(11)	18	11	P5(11)	18	69
C2(10)	19	133	P6(10)	19	23
J2(09)	20	182			
A7(09)	21	16			

Interestingly, the four highest ranked nodes are the same under both measures; in particular, the highest ranked node (L1(05)) corresponds to the main laboratory where the data are collected and it is clearly the most important actor in the network whether considered in a temporal or in a static way. On the other hand, the table reveals several differences worth exploring. From a first look, we see that, while the vertices highest ranked statically appear also among the highest ranked temporal ones, there are some nodes with insignificant static betweenness, whose temporal betweenness is extremely high. This is the case, for example, of nodes S1(10) and J1(06).

##### The case of node S1(10)

 To provide some interpretation for this behavior, we observe vertex S1(10) in more detail. This vertex corresponds to a poster presentation at a conference in 2010. We explore two insights. First, although S1(10) has a relatively low degree, it has a great variety of temporal connections. Only three out of ten incident edges of S1(10) are connected to actors that are born on and after 2010, and the rest of the neighbors appear in different times, accounting for at least one neighbor appearing each year for which the data are collected. This helps the node to operate as a temporal bridge between different time instances and to perhaps act as a knowledge mobilization accelerator.

Second, S1(10) is close to the center of the only static community present in [2010–2011] and it is connected to the two most important vertices in the network. The existence of a single dense community, and the proximity to two most productive vertices can explain its negligible static centrality value: while still connecting various vertices S1(10) is not the shortest connector, and its betweenness value is thus low. However, a closer temporal look reveals that it plays an important role as an interaction bridge between all the actors that appear in 2010 and later, and the ones that appear earlier than 2010. This role remained invisible in static analysis and only emerges when we pay attention to the time of appearance of vertices and edges. On the basis of these observations, we can interpret S1(10)’s high temporal betweenness value as providing a fast bridge from vertices created earlier and those appearing later in time. This might indicate reasons for further study of the importance of poster presentations that can blend tacit and explicit knowledge mobilization in human–poster presentation–human relations during conferences, and continue into future mobilization with new non-human actors as was the case for S1(10).

##### The case of node J1(06)

J1(06), the *Journal of Neurochemistry*, behaves similarly to S1(10) with its high temporal and low static rank. As opposed to S1(10), this node is introduced very early in the network (2006); however, it is only active (i.e., has new incident edges) in 2006 and 2007. It has only three neighbors, A1(06), A3(07), and C1(07), all highly ranked vertices statically (A1(06), A3(07)), or temporally (C1(07)). Since its neighboring vertices are directly connected to each other or in close proximity of two hops, J1(06) fails to act as a static short bridge among graph entities. However, its early introduction and proximity to the most prominent knowledge mobilizers helps it become an important temporal player in the network. This is because temporal journeys overlook geodesic distances and are instead concerned with temporal distances for vertices. These observations might explain the high temporal rank of J1(06) in the knowledge mobilization network.

#### A finer look at foremost betweenness

A key question is whether the birth-date of a node is an important factor influencing its temporal betweenness. To gain insights, we conducted a finer temporal analysis by considering $TB^ \mathcal {T}_\mathcal{F}$ for all possible birth-dates, i.e., for $\mathcal {T}=[x,2011],\forall x\in \{2005,2006,2007,2008,2009,2010,2011\}$. This allowed us to observe how temporal betweenness varies depending on the considered birth-date.

Before concentrating on selected vertices (statically or temporally important with at least one interval), and analyzing them in more detail, we briefly describe a temporal community detection mechanism that we employ in analysis.

##### Detection of temporal communities

**Fig. 2:**
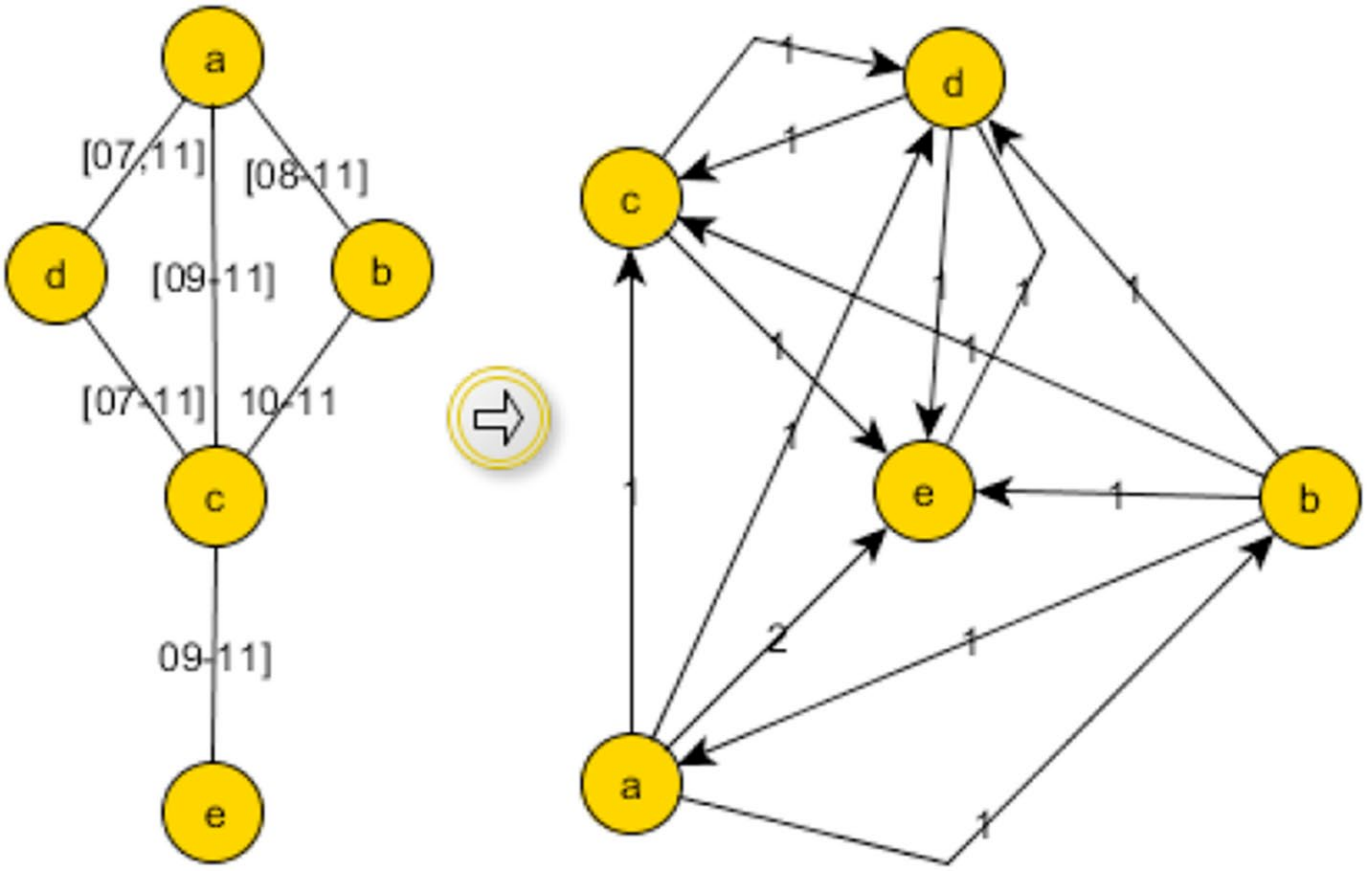
Transformation of a TVG into a journey graph

According to Tantipathananandh et al. [27], accurately detecting communities in TVGs is an NP-hard and APX-hard task. Tantipathananandh et al. [27] used a heuristic to approximate the community detection for a more efficient algorithm. However, when the number of nodes in a dense graph exceeds double digits, the algorithm becomes computationally unfeasible to run. To the best of our knowledge, the only other work that attacked the community detection problem in TVGs is [39], where the problem is tackled by transforming the TVG into a series of static snapshot graphs with no repeated nodes in snapshots, and by incrementally detecting and adding to communities. While the complexity of the algorithm is not provided, it immediately proves inapplicable to our problem as it (a) works only on series of snapshots with no repetition and (b) includes aging factor in calculations. Thus, we take an approach similar to the one proposed in [27], by only focusing on approximating the communities for the purpose of this research. To do that, we first transform our TVG into a static weighted directed graph (the *journey graph*), which gives a rough indication of the foremost journeys of the actual TVG. We then use the journey graph as input to an existing community detection algorithm, designed for weighted graphs [40]. More precisely, given a TVG $G= (V,E)$, we construct the journey graph of G, $J(G) = (V,E')$ as follows: the nodes of *J*(*G*) correspond to the original nodes of *G* and $(x,y) \in E'$ if there exists at least a foremost journey between *x* and *y* in *G*. The weight associated to edge $(x,y) \in E'$ is equal to the number of foremost journeys between *x* and *y* in *G*. An example of this construction is shown in Fig. 2.

Note that Knowledge-Net, over time, creates only one connected component, but the community analysis of the Knowledge-Net graph results in 14 communities. The largest community consists of almost 39% of nodes and is centered around L1(05). Given the large number of the nodes belonging to communities and the low number of detected communities, it is clear that some of the central nodes share communities with each other.

##### The case of node P1(06)

This is a research project led by the principle investigator at L1(05). The project was launched in 2006 and its official institutional and funded elements wrapped up in 2011. Data in Table 3 support that P1(06) has similar temporal and static ranks with regard to its betweenness in lifetime [2005–2011]. One could conclude that the temporal element does not provide additional information on its importance and that the edges that are incident to P(06)-1 convey the same temporal and static flow. However, there is still an unanswered question on whether or not edges act similarly if we start observing the system at different times. Will a vertex keep its importance throughout the system’s lifetime?

**Fig. 3:**
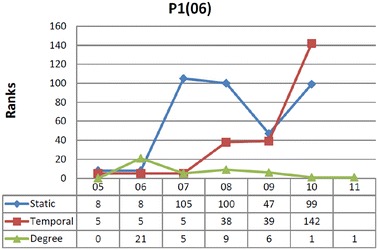
Comparison between different values for vertex P1(06). Ranks of the vertex in the last interval are not provided as both betweenness values are zero

The result of such analysis is provided in Fig. 3, where $TB^ \mathcal {T}_\mathcal{F} (P1(06))$ is calculated for each birth-date (indicated in the horizontal axis), with all intervals ending in 2011.

While both equally important during the entire lifetime [2005–2011] of the study, this project seems to assume a rather more relevant temporal role when observing the system in a lifetime starting in year 2007 (i.e., $\mathcal {T} =$ [2007–2011]), when its static betweenness is instead negligible. This seems to indicate that the temporal flow of edges incident to P1(06) appearing from 2007 on is more significant than the flow of the edges that appeared previously.

With further analysis of P1(06)’s neighborhood in [2007–2011], we can formulate technical explanations for this behavior. First, its direct neighbors also have better temporal betweenness than static betweenness. Moreover, its neighbors belong to various communities, both temporally and statically. However, looking at the graph statically, we see several additional shortest paths that do not pass through P1(06) (thus making it less important in connecting those communities). In contrast, looking at the graph temporally P1(06) acts as a mediator and accelerator between communities. More specifically, we observe that the connections P1(06) creates in 2006 contribute to the merge of different communities that appear only in 2007 and later. When observing within interval [2006–2011], we then see that P1(06) is quite central from a static point of view, because the appearance of time of edges does not matter, but, when observing it in lifetime [2007–2011], node P1(06) loses this role and becomes statically peripheral because the newer connections relay information in an efficient temporal manner.

In other words, it seems that P1(06) has an important role for knowledge acceleration in the period [2007–2011], a role that was hidden in the static analysis and that does not emerge even from an analysis of consecutive static snapshots. For research funders, revealing a research project’s potentially invisible mobilization capacity is relevant. Research projects can thus be understood beyond mobilization outputs and more in terms of networked temporal bridges to broader impact.

##### The case of node A3(07)

**Fig. 4:**
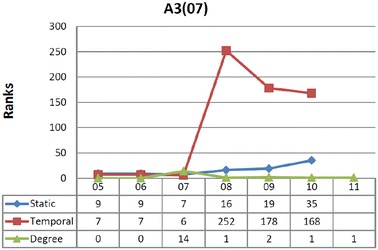
Comparison between different values for vertex A3(07). Ranks of the vertex in the last interval are not provided as both betweenness values are zero

Comparison between different values for vertex A3(07) are shown in Fig. 4, where ranks of the vertex in the last interval are not provided as both betweenness values are zero. The conditions for this node, a paper published in 2007, illustrate a different temporal phenomenon. Node A3(07) has several incident edges in 2007 (similarly to node P1(06)) when both betweenness measures are high. Peering deeper into the temporal communities formed around A3(07) is revealing: up to 2007, this vertex is two steps from vertices that connect two diffrent communities in the static graph. The situation radically changes, however, with the arrival of edges in 2008 that modify the structure of those communities, and push A3(07) to the periphery. The shift is dramatic from a temporal perspective because A3(07) loses its accelerator role where its temporal betweenness becomes negligible, while statically there is only a slight decrease in betweenness. The reason for a dampened decrease in static betweenness is that this vertex is close to the center of the static community, connecting peripheral vertices to the most central nodes of the network (such as L1(05) and H1(05)). It is mainly proximity to these important vertices that sustains A3(07)’s static centrality.

Such temporal insights lend further support to understanding mobilization through a network lens coupled with sensitivity to time. A temporal shift to the periphery for an actor translates into decreased potential for sustained mobilization.

### Invisible rapids and brooks

On the basis of our observations, we define two concepts to differentiate the static and temporal flow of vertices in knowledge mobilization networks. We call *rapids* the nodes with high foremost betweenness, meaning that they can potentially mobilize knowledge in a timelier manner, and *brooks* the ones with insignificant foremost betweenness. Moreover, we call *invisible rapids* those vertices whose temporal betweenness rank is considerably more significant than their static rank (i.e., the ones whose centrality was undetected by static betweenness), and *invisible brooks* the ones whose static betweenness is considerably higher than their temporal betweenness, meaning that these vertices can potentially be effective knowledge mobilizers, yet they are not acting as effectively as others due to slow or non-timely relations.

Invisible rapids and brooks can be present in different lifetimes as their temporal role might be restricted to some time intervals only; for example, as we have seen in the previous section, S1(10) and J1(06) are invisible rapids in $\mathcal{T} =$ [2005–2011], P1(06) is an invisible rapid in $\mathcal{T} =$ [2007–2011], and A3(07) is an invisible brook in $\mathcal{T} =$ [2008–2011]. Tables 4 and 5 indicate the major invisible rapids and brooks observed in *Knowledge-Net*.

**Table 4:** Major invisible rapids

Actor	Time	Temp. rank	Stat. rank	Type
P1(06)	[07–11]	5	105	Project
S1(10)	[05–11]	8	115	Poster
	[06–11]	8	113	
	[07–11]	7	115	
	[08–11]	5	104	
J1(06)	[05–11]	10	160	Journal
	[06–11]	10	154	
	[07–11]	10	223	
C1(07)	[05–11]	11	223	Citing publication
	[06–11]	11	220	
J2(09)	[06–11]	17	179	Journal
	[07–11]	16	182	
C2(10)	[05–11]	19	133	Citing poster
	[06–11]	16	132	
	[07–11]	15	133	

The presence of a poster presentation, a research project, two journals, and a conference publication among the invisible rapids supports that different types of mobilization actors can impact timely mobilization while not being as effective at creating short paths among entities for knowledge mobilization. In other words, they can play a role of accelerating knowledge mobilization, but to a concentrated group of actors.

**Table 5:** Major invisible brooks

Actor	Time	Stat. rank	Temp. rank	Type
J3(08)	[08–11]	9	117	Journal
	[09–11]	12	84	
C3(11)	[08–11]	10	191	Citing publication
	[09–11]	15	153	
C4(11)	[08–11]	15	105	Citing publication
H2(05)	[06–11]	16	118	Researcher
	[07–11]	15	134	
A3(07)	[08–11]	16	187	Publication
C5(07)	[08–11]	18	158	Citing publication

As for invisible brooks, we observe a journal (the *Biochemica et Biophysica Acta-Molecular Cell Research* (J3(08)), three papers (C3(11), C4(07), and C5(07)) that cite publications by the main laboratory in the study (L1(05)), a publication (A3(07)) mobilizing knowledge from members of L1(05), and a research assistant who worked on several research projects as an HQP. In comparison with invisible rapids, there is a wider variety in the type of mobilization actors that act as brooks which does not readily lend itself to generalization.

Interestingly, we see the presence of journals among invisible rapids and brooks. From our analysis, it seems that journals can hold strikingly opposite roles: on the one hand, they can contribute considerably to more timely mobilization of knowledge while not being very strong bridges between communities, while on the other hand they can play critical roles in bridging network communities, but at a slow pace. A brook, the journal *Biochemica et Biophysica Acta-Molecular Cell Research* (J3(08)), for example, helped mobilize knowledge in two papers for L1(05) (in 2008 and 2009), and is a journal in which a paper (in 2011) citing a L1(05) publication was also published. Given expected variability in potential mobilization for a journal, further research is needed to establish their roles in mobilization, whether these mobilization actors exist at both ends of the spectrum, or they have a neutral role in mobilization of knowledge.

In contrast, the presence of a research project as an invisible rapid might indicate meaningful observations that should be studied further. First, because when public funders invest in research projects as a mobilization actor, an implicit if not explicit measure of success is timely mobilization with potential impact inside and outside of academia [19]. Ranking as a rapid (for a mobilization actor) is one measure that could therefore help funding agencies monitor and detect temporal change in mobilization networks. Second, a research project as rapid might be meaningful because by its very nature a research project can help accelerate mobilization for the full range of mobilization actors, including other research projects. As such, it is not surprising that they can become temporal conduits to knowledge mobilization in all of its forms.

## Conclusions

In this paper, we proposed the use of a temporal betweenness measure (foremost betweenness) to analyze a knowledge mobilization network that had been already studied using classical “static” parameters. Our goal was to see the impact on the perceived static central nodes when employing a measure that explicitly takes time into account. We observed interesting differences. In particular, we witnessed the emergence of invisible rapids: nodes whose static centrality was considered negligible, but whose temporal centrality appears relevant. Our interpretation is that nodes with high temporal betweenness contribute to accelerate mobilization flow in the network and, as such, they can remain undetected when the analysis is performed statically. We conclude that foremost betweenness is a crucial tool to understand the temporal role of the actors in a dynamic network, and that the combination of static and temporal betweenness is complementary to provide insights into their importance and centrality.

The algorithm proposed in this paper to compute foremost betweenness constitutes a deterministic solution and its running time can be exponential in the worst case, which makes it applicable only on very small-scale networks. Since counting all foremost journeys in a graph is a #P-complete problem, such a high cost is inevitable for any deterministic solution. An open interesting direction is the design of approximate solutions, feasible for large networks.

Temporal network analysis as performed here is especially pertinent for KM research that must take time into account to understand academic research impact beyond the narrow short-term context of academia. Measures of temporal betweenness, as studied in this paper, can provide researchers and funders with critical tools to more confidently investigate the role of specific mobilization actors for short- and long-term impact within and beyond academia. The same type of analysis could clearly be beneficial when applied to any other temporal context.

In conclusion, we focused here on a form of temporal betweenness designed to detect accelerators. This is only a first step toward understanding temporal dimensions of social networks; other measures are already under investigation.
